# Mass Spectrometry Based Imaging Techniques for Spatially Resolved Analysis of Molecules

**DOI:** 10.3389/fpls.2013.00089

**Published:** 2013-04-19

**Authors:** Andrea Matros, Hans-Peter Mock

**Affiliations:** ^1^Leibniz Institute of Plant Genetics and Crop Plant ResearchGatersleben, Germany

**Keywords:** mass spectrometry, imaging, secondary metabolites, primary metabolism, proteins, peptides, metabolite distribution

## Abstract

Higher plants are composed of a multitude of tissues with specific functions, reflected by distinct profiles for transcripts, proteins, and metabolites. Comprehensive analysis of metabolites and proteins has advanced tremendously within recent years, and this progress has been driven by the rapid development of sophisticated mass spectrometric techniques. In most of the current “omics”-studies, analysis is performed on whole organ or whole plant extracts, rendering to the loss of spatial information. Mass spectrometry imaging (MSI) techniques have opened a new avenue to obtain information on the spatial distribution of metabolites and of proteins. Pioneered in the field of medicine, the approaches are now applied to study the spatial profiles of molecules in plant systems. A range of different plant organs and tissues have been successfully analyzed by MSI, and patterns of various classes of metabolites from primary and secondary metabolism could be obtained. It can be envisaged that MSI approaches will substantially contribute to build spatially resolved biochemical networks.

## Background

Spatially resolved analysis of metabolites and proteins has become feasible within recent years by the development of mass spectrometry imaging (MSI) strategies. Development and application of these techniques has been pioneered in medicinal and pharmacological research. MS imaging allowed the detection of novel clinical markers for better diagnosis of cancer tissues and to follow the spatial-temporal patterns of drug molecules used for pharmacological studies (Rauser et al., 2010; Schwamborn et al., 2010).

The application of MSI strategies has now also been introduced in plant research. As higher plant organs are composed of a multitude of tissues, information on the spatial distribution of proteins and metabolites will be essential to generate improved models of metabolism and to assign biochemical functions of specific tissues. Sample preparation is a major bottleneck for successful MSI of plant tissues. The majority of the MSI studies on plants published to date have addressed the spatial distribution of certain classes of metabolites or of peptides or small proteins. Application of MSI of large proteins still provides considerable difficulties in practice. In the paper current strategies to address these limitations in MSI of plant proteins will be discussed. The particular advantages of MSI set-ups such as matrix-assisted laser desorption ionization (MALDI) MS or desorption electrospray ionization (DESI) MS will be highlighted. Approaches complementary to the MSI strategies will be briefly mentioned, in particular the use of laser microdissection of defined areas of tissue sections. Finally, data evaluation and integration into modeling approaches will be addressed.

## MSI Sample Preparation

Any sample preparation technique for MSI analyses aims on keeping the lateral resolution and the nature of the target molecules. According to the applied MSI technique it varies from just mounting a sample for surface analysis to delicate sectioning and matrix application procedures. Some recent publications provide detailed protocols on plant sample preparation for small molecule MSI (Peukert et al., 2012), as well as for protein MSI (Grassl et al., 2011). Strategies for optimized sectioning have been proposed such as varying conditions for sample freezing (e.g., dry ice for water rich samples), section mounting (e.g., embedding in water or gelatin for tiny or flat samples), variation of section thickness (10- to 35-μm), or section drying. Washing steps applied on the sectioned samples will impact on the classes of molecules retained on the tissue surface. Small molecules can be removed to improve imaging of peptides and proteins (Kaspar et al., 2011). Also, the choice of matrix and application strategy strongly influences the sort of molecular species which are ionized and the lateral resolution of the MS images. An increasing number of matrices for various applications have been recently explored and the interested reader is kindly referred to relevant publications (Svatos, 2010; Greer et al., 2011; Kaspar et al., 2011). For reproducible matrix application the most widely used deposition techniques are spraying with a simple airbrush and the use of a dedicated instrument to obtain vibrational vaporization. In our hands vibrational vaporization using a commercial device (ImagePrep, Bruker Daltonics, Germany) is most suitable to adjust optimal spraying and drying times, such as needed for multiple matrix layers and tryptic digestion on tissue sections.

## MSI Techniques

All MSI techniques represent surface analysis techniques which are based on desorption and ionization of molecules followed by their subsequent MS data recording (Chaurand, 2012). The most common technique applied for MSI of metabolites and peptides is MALDI MSI, involving the application of a suitable matrix substance on the surface (Caprioli et al., 1997). Other common ionization processes are DESI utilizing a solvent stream (Takáts et al., 2004), secondary ion mass spectrometry (SIMS) making use of an ion beam (Vickerman, 2011), and laser ablation electrospray ionization (LAESI) (Nemes and Vertes, 2012). As DESI and LAESI techniques operate exclusively under atmospheric pressure (AP), sample preparation and associated issues are minimized. Most SIMS and MALDI sources operate in a vacuum chamber, which leads to the loss of volatile compounds and requires careful sample preparation (Chughtai and Heeren, 2010; Kaspar et al., 2011). Cryosectioning is a common procedure to prepare tissue sections for subsequent MALDI MSI (please refer to section MSI Sample Preparation). The spatial resolution currently achieved differs between the various ionization techniques. The highest spatial resolution (<1 μ) of current instruments is achieved for SIMS. Due to high fragmentation and low ionization efficiency, the size of biological molecules detected by SIMS analysis is limited. Instead, MALDI MSI has been favored with a current limit for spatial resolution of about 10 μm (Lee et al., 2012). Spatial resolution in MALDI MSI is influenced by the laser spot size, but is also strongly dependent on matrix application. Formation of large matrix crystals will negatively impact the spatial resolution to be achieved for the sample (Svatos, 2010; Peukert et al., 2012). DESI and LAESI techniques can be applied when the surface chemistry of the sample itself is of interest. Due to the low input for sample preparation, these approaches are suitable of screening larger sample sets (Svatos, 2010).

Various commercial mass analyzers are available for MSI providing sufficient: (i) mass resolution, (ii) spatial resolution, and (iii) MS scan speed. However, selection of one or another technique remains a compromise as none of the available mass analyzers meets perfectly all criteria, e.g., high resolution mass spectrometer typically have slower scan speed (Lee et al., 2012). The recent implementation of tandem mass spectrometry has encouraged MSI applications in plant research by enabling the identification of metabolites and, via on tissue digestion, N-terminal peptide derivatization and CID tandem MS, by facilitating the identification of polypeptides (Horn et al., 2011; Lunsford et al., 2011; Muller et al., 2011).

## MSI of Small Molecules

Imaging of small molecules including different classes of primary and secondary metabolites are the most frequent applications to date within plant MSI (Burrell et al., 2007; Kaspar et al., 2011; Lee et al., 2012; Peukert et al., 2012). Studies with a focus on method development and technology application on metabolites that are readily accessible for analysis are still highest abundant among available publications. However, an increasing number of experimental applications became available recently and an overview is presented in Table 1. Differential distribution pattern have been evaluated for a number of molecular species, namely, lipids, amino acids, and sugars, as well as high abundant secondary metabolites, such as polyphenols, anthocyanins, alkaloids, and glucosinolates from a variety of plant species. In the following, we will describe a number of selected examples in more detail.

**Table 1:** **Published literature describing the application of MS imaging for the analysis of small molecules from plant material**.

Plant material	Applied technique	Applied matrix	Molecular species	Reference
***Arabidopsis thaliana***
Leaf	MALDI MSI	9-Aminoacridine	Glucosinolates	Shroff et al. (2008)
Leaf	MALDI MSI	Lithium DHB^1^	Neutral lipids	Vrkoslav et al. (2010)
Leaf	LDI MSI	None	Flavonoids	Hoelscher et al. (2009)
Flower parts	GALDI MSI	Colloidal silver/colloidal graphite	Epicuticular lipids	Jun et al. (2010)
Flower petal	GALDI MSI	Colloidal graphite	Flavonoids	Perdian et al. (2010)
Root	GALDI MSI	Colloidal silver/colloidal graphite	Alkyl esters of coumarate, caffeate and ferulate, sterols	Jun et al. (2010)
Stem/flower/leaf	GALDI MSI	Colloidal graphite	Flavonoids, cuticular waxes	Cha et al. (2008)
Leaf/flower	GALDI MSI	Colloidal silver	Epicuticular waxes	Cha et al. (2009)
***Triticum aestivum***
Stem	MALDI MSI	α-CHCA^2^	Oligosaccharides	Robinson et al. (2007)
Grain	MALDI MSI	α-CHCA, 9-aminoacridine	Amino acids, sugars, sugar phosphates	Burrell et al. (2007)
***Hordeum vulgare***
Grains	MALDI MSI	DHB	Lipids	Peukert et al. (2012)
Leaf	DESI MSI	None	Hydroxynitrile glucosides	Li et al. (2011)
***Oryza sativa***
Grain	MALDI MSI	DHB	Lipids, γ-oryzanol, phytic acid	Zaima et al. (2010)
***Hypericum perforatum***
Leaf/flower parts	LDI MSI	None	Naphthodianthrones	Hoelscher et al. (2009)
Leaf/petal	DESI MSI	None	Secondary metabolites	Thunig et al. (2011)
***Solanum melongena***
Fruit	MALDI MSI	DHB	γ-Aminobutyric acid, amino acids, sugars	Goto-Inoue et al. (2010)
***Nicotiana tabacum***
Leaf	IR AP^3^ MALDI MSI	None	Phenolics, alkaloids, oxylipins, sugars, among others	Ibanez et al. (2010)
Stem	MALDI MSI	DHB	Lipids	Peukert et al. (2012)
***Solanum tuberosum***
Tubers	MALDI MSI	DHB	Glycoalkaloids	Ha et al. (2012)
***Helianthus annuus***
Stem	MALDI MSI	α-CHCA	Nicosulfuron (pesticide)	Anderson et al. (2010)
***Glycine max***
Leaf/stem	MALDI MSI	α-CHCA/SA	Azoxystrobin (fungizide)/mesotrione (herbicide)	Mullen et al. (2005)
***Phoenix sp. (date palm tree)***
Leaf	MALDI MSI	Lithium DHB	Neutral lipids	Vrkoslav et al. (2010)
***strawberry/banana/grapes***
Fruits	IR AP MALDI MSI	None	Sugar monomers and oligomers, citric acid	Li et al. (2007)
***Gossypium hirsutum***
Seed/embryo	MALDI MSI	DHB	Lipids	Horn et al. (2012)
***Populus sp***.
Stem	MALDI MSI	DHB	Oligosaccharides, polysaccharides	Lunsford et al. (2011)
***Vaccinium ashei*(blueberry)**
Fruit	MALDI MSI	DHB	Anthocyanins	Yoshimura et al. (2012)
***Malus sp. (Golden delicious)***
Fruit	MALDI MSI	α-CHCA	Flavonoids, dihydrochalcones	Franceschi et al. (2012)
***Myristica malabrica(Lam)***
Fruit	DESI MSI	None	Alkaloids	Ifa et al. (2011)
***Stevia rebaudiana***
Leaf	DESI MSI	None	Diterpenes	Gray et al. (2009)

*^1^2,5-Dihydroxybenzoic acid*.

*^2^α-Cyano-4-hydroxycinammic acid*.

*^3^Infrared atmospheric pressure*.

Most analyses still rely on MALDI MSI techniques with variant matrices applied. For example MALDI MSI was applied to visualize the lipid species in cotton seed tissues (Horn et al., 2012). The comprehensive MSI study demonstrated distinct spatial patterns for molecular species of triacylglycerols and phosphatidylcholine. The MSI data set contained also information on a wide range of other lipid molecules, such as phosphatidylethanolamines, phosphatidic acids, sterols, and gossypol, supporting the wide applicability of the imaging approach (Horn et al., 2012). Also, non-uniform distribution of glucosinolates within *Arabidopsis* leaves was revealed by MALDI MSI (Shroff et al., 2008). Repeated spraying of the matrix 9-aminoacridine allowed the extraction of glucosinolates from the tissue beneath the surface. The major glucosinolates of the leaves were more abundant in tissues of the midvein and in the periphery when compared to the inner lamina. This pattern seemed to determine the feeding preference of insect larvae for the inner lamina (Shroff et al., 2008).

The application of colloidal graphite (GALDI) enabled the MSI of various small molecules in *Arabidopsis* surfaces and tissue sections (Cha et al., 2008). When analyzing leaf surfaces, very long chain fatty acids (C26, C28, and C30) were observed. Mass signals diagnostic for flavonoids, which localized within the cells, were only observed at positions were the leaf samples were damaged. When the surface epicuticular waxes were removed by dipping the leaves briefly into chloroform, the signals for fatty acids were strongly reduced. Instead, ions corresponding to kaempferol derivatives became apparent. MSI of flower leaves and stem sections also demonstrated heterogeneous distribution of flavanoids in this organs (Cha et al., 2008).

Matrix free UV-laser desorption/ionization (LDI) MSI at the single cell level provided information on the spatial distribution of UV-absorbing secondary metabolites for *Arabidopsis thaliana* (kaempferol derivatives) and *Hypericum* species (phloroglucinols and naphthodianthrones) (Hoelscher et al., 2009). The authors thoroughly confirmed and complemented the MSI data by analysis of isolated glands obtained through microdissection (Hoelscher et al., 2009).

Barley leaf tissue was subjected to MSI using direct and indirect DESI (Li et al., 2011) revealing a homogeneous distribution of hydroxynitrile glucosides. For direct DESI, the epidermis was stripped off and its back was analyzed. Indirect DESI was performed on imprints from intact leaves and of peeled epidermal strips using porous teflon. The indirect approach allowed relative quantification of these compounds in three divergent barley cultivars, namely Mentor, Golden Promise, and Emir (Li et al., 2011).

Only recently approaches have been published that utilize a combination of MALDI MS for imaging and high resolution MS for identification. MALDI MSI in combination with linear ion trap MS was required to study the distribution of the complex polymers cellulose and hemicelluloses in poplar tissue (Lunsford et al., 2011). MS spectra alone provided an even distribution of cellulose and hemicelluloses ions; however, when plotting characteristic fragment ions obtained by MS/MS, quite contrasting images were obtained. The authors concluded that tandem MS is necessary to separate isobaric species in order to accurately annotate wood tissue MS images. They also observed reduced background in the MS/MS experiments, which improved the signal-to-background ratio in the image analysis (Lunsford et al., 2011).

## MSI Analysis of Peptide and Protein Patterns

Mass spectrometry-based imaging of proteins is of particular interest for biomedical research. However, the mass range for sensitive detection of proteins in tissue sections is limited. Identification of proteins in imaging experiments is still a challenging task. On tissue digestion using proteolytic enzymes is possible; conversely this procedure increases the complexity of the MS spectra considerably. This fact necessitates high accurate mass measurements (Schober et al., 2011). Another challenge is to keep spatial resolution during tryptic digestion of the proteins on the tissue surface. A protocol has been developed which allowed to achieve a spatial resolution of 50 μm (Schober et al., 2012).

So far, few reports describe the MSI analysis of peptides from plant tissues. *In situ* MALDI MS analysis determined the structure of a modified 12-amino acid peptide (MCLV3), which was derived from a conserved motif in the CLV3 sequence in *Arabidopsis* callus (Kondo et al., 2006). The spatial distribution of cyclotide peptides was analyzed by MALDI MSI in *Petunia* leaves (Poth et al., 2012). Cyclotides represent a family of plant peptides and are characterized by a structural feature called the cycline cystine knot. Several reports suggest a role of cyclotides in plant defense. The non-uniform distribution of cyclotides in *Petunia* leaves would be consistent with such a role (Poth et al., 2012). Additionally, discriminative peptides in barley grain sections were highlighted as examples in a recent review (Kaspar et al., 2011).

Most protein MSI studies to date are from the field of medicine (Caprioli et al., 1997; Yanagisawa et al., 2003; Schwartz et al., 2005; Goodwin et al., 2008). Spatial distribution of proteins can be used as markers for diagnosis of diseases and to better differentiate between diseased and healthy tissues. Unknown proteins can serve as valuable markers and assist diagnosis and disease treatment. Their identification will be necessary to build a biochemical network representing the molecular events underlying the development of the disease (Mascini and Heeren, 2012). Targeted MSI for proteins was recently performed by means of the combination of immunohistochemistry with MALDI MSI using single chain fragment variable recombinant antibodies (Thiery et al., 2012).

The first report on protein MSI for plants described the detection and identification of the allergenic lipid transfer protein Pru p3 in the peel of the peach fruit by means of electrospray MS identification and MALDI MSI (Cavatorta et al., 2009). Method development for MALDI MSI of proteins was recently published, encouraging the wider application of protein MSI in plant tissues (Grassl et al., 2011). Main constraints for MSI of intact proteins present the rather low protein abundance, the high water content, and the rigid cell walls and abundant air spaces in many plant tissues, resulting mainly in challenging sample preparation. As for the presented soybean sample, the authors propose to prepare and freeze the sample using dry ice immediately after collection to preserve morphology and minimize protein degradation through proteolysis and to avoid breakage and cracking as observed during shock freezing by liquid nitrogen. Optimal section thickness for soybean seedlings has been found to be 10- to 15-μm from frozen sections. However embedding in gelatin showed an improvement in localization, lateral resolution, and reproducibility, with some loss in signal-to-noise. Soaking tissues in sucrose, and thus filling of air spaces between cells, dramatically improves cryosectioning and the lateral resolution during imaging. A wash with ice-cold 2-propanol enabled the fixation of proteins, and removed lipids and salts substantially. Complete drying of the tissue sections was also shown to be important in order to preserve protein localization. Best reproducibility in ion intensity as well as spatial resolution was observed when using sinapinic acid (SA) as a matrix. Matrix application was performed by means of vibrational spray (Image Prep, Bruker Daltonics, Germany) with an optimized protocol for spraying and drying cycles. For MALDI MSI measurement the authors suggested to adjust a resolution of 30–100 μm for the laser raster points. In addition, the authors comprehensively reviewed approaches enabling the identification of MSI protein targets either “on tissue” or by extraction procedures aiming to conserve the spatial localization (Poth et al., 2012). However, none of these identification approaches has been successfully applied to plants so far.

## Evaluation of MSI Data Sets

Analysis of multiple tissue sections at high spatial resolution necessarily generates large data sets providing challenges for the subsequent data mining. A number of imaging software packages are available, both open source (e.g., BioMap, see http://www.maldi-msi.org/) as well as commercial solutions (Kaspar et al., 2011). Still the visualization as well as the statistical treatment of large data sets requests further developments. Identification of unknown compounds requires high resolution mass spectrometry. Frequently, additional efforts are necessary to annotate compounds of interest, such as targeted analysis of micro-dissected materials or other complementary approaches. Software capable of handling three-dimensional datasets will be another essential tool for visualization.

## Further Development of MS Imaging

Spatial resolution of current instrumentation for LDI/MALDI MSI is restricted to 10–20 μm. MSI at cellular and in particular sub-cellular resolutions requires improvements in the techniques. Recently, the Caprioli group has developed transmission geometry MALDI MS allowing submicron spatial resolution (Zavalin et al., 2012). As a feature, the transmission geometry vacuum ion source enabled to irradiate the back of the sample with the laser beam. The development of this laser optics together with an adjusted sample preparation protocol allowed sufficient sensitivity of the instrument also at submicron spatial resolution (Zavalin et al., 2012). Further implementation of MSI will benefit from such specific developments, but also from the overall advances still seen in bio-analytical mass spectrometry instrumentation.

## Conclusion

Mass spectrometry imaging has recently been introduced into plant sciences mostly focused on the spatial distribution of low molecular weight compounds, including primary and secondary plant metabolites as well as cyclic peptides (Table 2). These studies will encourage extension of the approach toward other plants systems and applications. Sample preparation, selection of matrix substances and application of the matrix are critical to obtain images of sufficient quality using MALDI MSI. DESI MSI together with a number of other aforementioned imaging approaches provides strategies with complementary applications. Most promising are future developments in tandem MS technologies, such as combining MALDI MSI with high resolution MS for identification, and thus enabling the correlation of molecular distribution pattern to particular molecular networks and tissue function, and the quantitation of differential distributions. The studies already published will guide the further implementation of tandem MSI techniques for plant samples and extend the range of possible applications.

**Table 2:** **Schematic representation of the current status of MSI in plant science**.

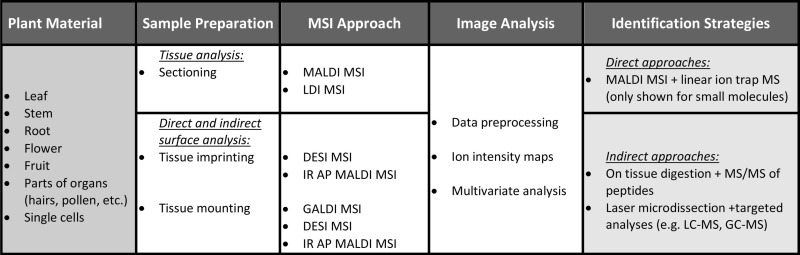

*For molecular species targeted by the different approaches please refer also to Table 1*.

## Conflict of Interest Statement

The authors declare that the research was conducted in the absence of any commercial or financial relationships that could be construed as a potential conflict of interest.
